# Long-term follow-up of overall survival for cabozantinib versus everolimus in advanced renal cell carcinoma

**DOI:** 10.1038/s41416-018-0061-6

**Published:** 2018-03-26

**Authors:** Robert J. Motzer, Bernard Escudier, Thomas Powles, Christian Scheffold, Toni K. Choueiri

**Affiliations:** 1 Memorial Sloan Kettering Cancer Center, New York, NY 10075 USA; 2 Institut Gustave Roussy, Villejuif, 94805 France; 3 Barts Cancer Institute, Queen Mary University of London, London, EC1M 6BQ UK; 4 Exelixis, Inc, South San Francisco, CA 94080 USA; 5 Dana-Farber Cancer Institute, Boston, MA 02215 USA

**Keywords:** Renal cell carcinoma, Targeted therapies

## Abstract

**Background:**

In the phase 3 METEOR trial (NCT01865747), cabozantinib significantly improved progression-free survival, overall survival, and objective response rate compared with everolimus in patients with advanced renal cell carcinoma (RCC) after prior antiangiogenic therapy. A statistically significant improvement in overall survival was observed at a second interim analysis with 320 recorded deaths.

**Methods:**

658 patients with advanced RCC who had received at least one prior VEGFR tyrosine kinase inhibitor were randomised 1:1 to cabozantinib (60 mg daily) or everolimus (10 mg daily). Survival follow-up continued to reach the 408 deaths that were pre-specified for the final analysis.

**Results:**

With 430 deaths (198 for cabozantinib and 232 for everolimus), median overall survival was 21.4 months with cabozantinib and 17.1 months with everolimus (HR 0.70, 95% CI 0.58–0.85; *P* = 0.0002). Safety profiles of cabozantinib and everolimus were consistent with those reported previously.

**Conclusions:**

Cabozantinib significantly improved overall survival compared with everolimus in previously treated patients with advanced RCC with consistent results after long-term follow-up.

## Introduction

Cabozantinib is an oral inhibitor of tyrosine kinases including MET, vascular endothelial growth factor (VEGF) receptors, and AXL.^1^ Cabozantinib is approved for the treatment of patients with advanced renal cell carcinoma (RCC) after prior antiangiogenic therapy based on results of the phase 3 METEOR trial showing significant improvements in progression-free survival, overall survival, and objective response rate compared with everolimus.^2, 3^ At the second interim analysis with 320 (78%) of the 408 planned events and a minimum follow-up of 13 months, the null hypothesis of no difference in overall survival was rejected. Differences in overall survival were statistically significant with a median of 21.4 months with cabozantinib versus 16.5 months with everolimus (hazard ratio [HR] 0.66, 95% CI 0.53–0.83; *P* = 0.0003). Subgroup analyses of overall survival were consistent with results for the overall population. We report results for overall survival and updated safety after follow-up was continued to reach the number of deaths pre-specified for the final analysis.

## Materials and methods

METEOR is a randomised, open-label, phase 3 trial with patients enrolled at 173 centres in 26 countries.^2, 3^ Eligible patients were 18 years of age or older with advanced or metastatic clear-cell RCC and measurable disease per Response Evaluation Criteria in Solid Tumors (RECIST version 1.1).^4^ Patients must have received at least one prior VEGFR tyrosine kinase inhibitor (TKI) and must have progressed within 6 months of their most recent VEGFR TKI and within 6 months of randomisation. A Karnofsky performance status of at least 70 was required.

Patients were randomised 1:1 to cabozantinib (60 mg once daily) or everolimus (10 mg once daily). Randomisation was stratified by Memorial Sloan Kettering Cancer Center (MSKCC) risk group and number of prior VEGFR TKIs (1 versus 2 or more). Dose reduction levels to manage adverse events were 40 and 20 mg for cabozantinib and 5 and 2.5 mg for everolimus. To maximise the ability to evaluate the effect of study treatment on overall survival, treatment crossover was not allowed in the study. The study was conducted according to the Good Clinical Practice guidelines and the Declaration of Helsinki. The protocol was approved by the institutional review board or ethics committee at each centre, and written informed consent was obtained for all patients.

The primary endpoint of progression-free survival and secondary endpoints of overall survival and objective response rate and safety have been reported previously.^2, 3^ After determination of the primary endpoint, tumour scans were no longer centrally collected nor analysed in aggregate. Safety, including adverse events, was evaluated every 2 weeks for the first 8 weeks and every 4 weeks thereafter until treatment discontinuation. Adverse events were graded according to Common Terminology Criteria for Adverse Events version 4.0. Patients were followed for overall survival every 8 weeks. Information on subsequent anticancer therapy was also collected.

Overall survival was analysed in all 658 randomised patients. For overall survival, 408 deaths were required to detect a HR of 0.75 (80% power, two-sided α = 0.04) with a hypothesised improvement in median survival from 15 months to 20 months and one planned interim analysis to be conducted at the time of the primary endpoint analysis of progression-free survival. The first planned interim analysis did not meet the criteria for significance defined by the Lan-DeMets O’Brien-Fleming alpha spending function. However, the criteria for significance for rejection of the null hypothesis was met at a second interim analysis conducted with a prospectively defined cutoff date of 13 December 2015 and a minimum follow-up of 13 months (*P* = 0.0003; critical *P*-value ≤ 0.0163).^2, 3^ Follow-up was continued to reach the 408 deaths pre-specified for the final analysis to collect long-term survival data.

Hypothesis testing of overall survival was performed using the stratified log-rank test with the randomisation stratification factors. Subgroup analyses of overall survival used all randomised patients and were pre-specified; HRs for subgroup analyses are unstratified and confidence intervals are considered descriptive.

## Results

A total of 658 patients were randomised from August 2013 to November 2014 (330 to cabozantinib and 328 to everolimus). Baseline characteristics were balanced between treatment arms.^2, 3^ The majority of patients were male (75%) with a median age of 62 years. Forty-six percent of patients were in the favourable risk category, 42% in the intermediate risk category, and 13% in the poor risk category as defined by MSKCC prognostic criteria for RCC.

**Fig. 1:**
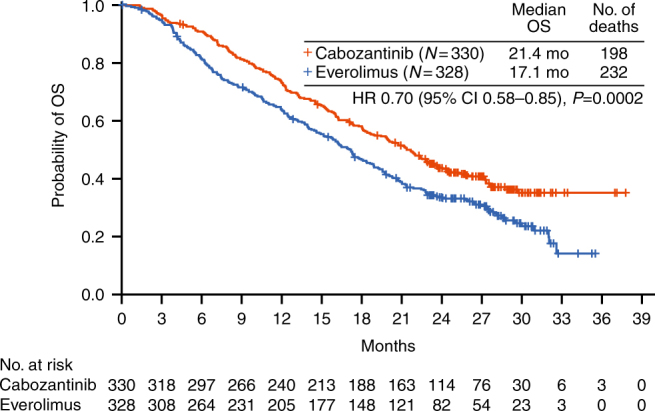
Overall survival through 2 October 2016. HR hazard ratio, OS overall survival

**Fig. 2:**
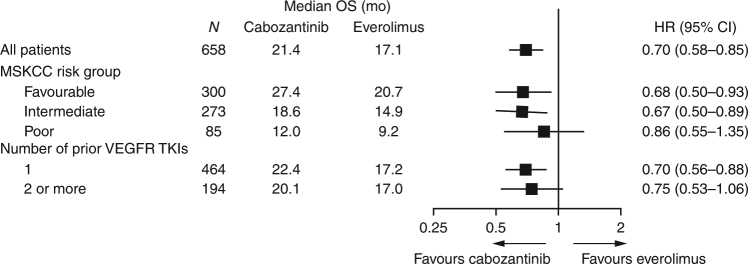
Forest plots of overall survival through 2 October 2016. All randomised patients were included in the analyses. All HRs are unstratified except for the analysis in all patients. HR hazard ratio, MSKCC Memorial Sloan Kettering Cancer Center, OS overall survival, TKI tyrosine kinase inhibitor, VEGFR vascular endothelial growth factor receptor

As of the 2 October 2016 data cutoff for overall survival, 11% (36/330) of patients in the cabozantinib group and 2.4% (8/328) of patients in the everolimus group remained on study treatment. The minimum follow-up was 22 months, median follow-up was 28 months (IQR 25, 30), and 430 deaths were recorded (198 for cabozantinib and 232 for everolimus). Analysis of overall survival showed a significant improvement for cabozantinib compared with everolimus: median overall survival was 21.4 months with cabozantinib and 17.1 months with everolimus (HR 0.70, 95% CI 0.58–0.85; *P* = 0.0002) (Fig. 1). Overall survival landmark estimates for cabozantinib and everolimus were 44% versus 34% at 24 months and 35% versus 25% at 30 months. Overall survival analyses of subgroups defined by stratification factors were consistent with results for the overall population (Fig. 2). Use of systemic subsequent anticancer therapy was 57% versus 63% for cabozantinib versus everolimus and included VEGFR TKIs (28% for cabozantinib versus 50% for everolimus), everolimus (33% versus 5%), PD-1 checkpoint inhibitors (14% versus 16%, with 13% versus 15% receiving nivolumab), and cabozantinib (1% versus 4%).

The safety population consisted of 331 cabozantinib-treated patients and 322 everolimus-treated patients. As of the 2 October 2016 data cutoff, the median duration of exposure was 8.4 months (range, 0.3–37.0) for cabozantinib-treated patients versus 4.4 months (range, 0.2–32.2) for everolimus-treated patients. Dose reductions due to adverse events were implemented in 64% of cabozantinib-treated patients and 25% of everolimus-treated patients. The median average daily dose was 42.8 mg for cabozantinib and 9.1 mg for everolimus. Discontinuation due to adverse events occurred for 43 (13%) cabozantinib-treated patients and 35 (11%) everolimus-treated patients.

All patients in the cabozantinib group and all but one patient in the everolimus group experienced an adverse event of any grade irrespective of causality; grade 3 or 4 adverse events were experienced by 71% of cabozantinib-treated patients and 61% of everolimus-treated patients (Table 1). Grade 5 events occurred for 31 (9%) cabozantinib-treated patients and 26 (8%) everolimus-treated patients. One grade 5 adverse event was treatment-related in the cabozantinib group (death), and two were treatment-related in the everolimus group (aspergillus infection and pneumonia aspiration).

**Table 1:** All causality grade 3 or 4 adverse events

	Cabozantinib (*N* = 331)	Everolimus (*N* = 322)
Any adverse event, *n* (%)	236 (71)	196 (61)
Hypertension	51 (15)	12 (4)
Diarrhoea	44 (13)	8 (2)
Fatigue	36 (11)	24 (7)
PPE	28 (8)	3 (1)
Anaemia	22 (7)	55 (17)
Hypokalaemia	16 (5)	6 (2)
Hypomagnesaemia	16 (5)	0
Hyponatraemia	16 (5)	8 (2)
Nausea	16 (5)	1 (<1)
Asthaenia	15 (5)	8 (2)
Hyperglycaemia	3 (1)	16 (5)

Adverse events that occurred at ≥5% in either treatment arm are summarised. Patients are counted once at the highest grade for each preferred term. The severity of adverse events was graded according to the National Cancer Institute Common Terminology Criteria for Adverse Events (version 4.0).

*PPE* palmar-plantar erythrodysesthesia

## Discussion

This study reports long-term survival data with a minimum follow-up of 22 months from the pivotal phase 3 METEOR trial of cabozantinib versus everolimus in patients with previously treated advanced RCC. Results show a significant improvement in overall survival for cabozantinib compared with everolimus that was consistent with the earlier analysis in which the null hypothesis was rejected. With 9 additional months of follow-up, median survival was 21.4 months with cabozantinib and 17.1 months with everolimus (HR 0.70) compared with 21.4 months with cabozantinib and 16.5 months with everolimus (HR 0.66) at the earlier analysis.^2^ Subgroup analyses of overall survival based on the stratification factors were consistent with results for the overall population showing a survival benefit with cabozantinib compared with everolimus, similar to previous results.^2^

The PD-1 checkpoint inhibitor nivolumab has also shown a benefit in overall survival in a similar patient population when compared with everolimus.^5^ In the nivolumab study, long-term survival data at 3 years showed a HR of 0.74 (95.45% CI 0.63–0.88).^6^

A possible limitation of analysing survival outcomes with longer follow-up is that increased use of subsequent systemic therapy may confound the results. The percent of patients who received subsequent systemic therapy was similar in the two treatment groups; however, the type of subsequent therapy received differed. Patients in the cabozantinib group more frequently received subsequent everolimus, while patients in the everolimus group more frequently received subsequent therapy with VEGFR TKIs. Importantly, subsequent therapy with the PD-1 checkpoint inhibitor nivolumab, which has demonstrated prolongation of overall survival in this patient population,^5^ was similar in both treatment groups.

The safety profiles of cabozantinib and everolimus were consistent with those reported previously,^2^ with similar reported incidences of adverse events, dose reductions, and discontinuations due to adverse events. The similar results reflect both the reduced number of patients on study treatment and continued tolerability for those remaining on treatment.

Cabozantinib significantly improved overall survival compared with everolimus in previously treated patients with advanced RCC with a consistent benefit after long-term follow-up.
